# Comparison of analgesic therapies in traumatic vertebral compression fractures: Vertebroplasty and kyphoplasty versus thermal facet ablation—a prospective randomized trial

**DOI:** 10.1016/j.xnsj.2026.100954

**Published:** 2026-10-02

**Authors:** Tamás Tóth, Kerim Watfa, Márton Balázsfi, Pál Barzó

**Affiliations:** Department of Neurosurgery, Faculty of Medicine, University of Szeged, Semmelweis St 6, Szeged 6725, Hungary

**Keywords:** Vertebroplasty, Kyphoplasty, Thermal facet ablation, Vertebral compression

## Abstract

**Background:**

Vertebral augmentation techniques are common for painful vertebral compression fractures (VCFs). Radiofrequency thermal facet ablation (RFA) is a potential alternative analgesic therapy, but comparative data remains limited.

**Methods:**

In this single-center, prospective randomized controlled trial, patients with painful VCFs refractory to conservative treatment were randomized to vertebral augmentation or RFA. Pre- and postoperative Visual Analogue Scale (VAS) for axial pain and procedure-related complications were evaluated over a 6-month period.

**Results:**

Among 535 patients completing follow-up (290 augmentation, 245 RFA), both arms provided robust analgesia. Mean VAS reduction at 6 months was −5.3 (95% CI: −5.6 to −5.0) in the RFA group and −5.6 (95% CI: −5.9 to −5.3) in the augmentation group. The augmentation group had an 8% cement leakage rate (23 cases), with 2 patients (0.5%) requiring decompression, and a mean length of stay (LOS) of 1.4 days. The RFA arm demonstrated a zero-complication rate and allowed for same-day discharge (LOS 0.0 days).

**Conclusions:**

RFA and vertebral augmentation are highly effective at providing significant pain relief for VCFs, with comparable reductions in pain scores. With a zero-complication rate and outpatient feasibility, RFA is a safe and resource-efficient first-line alternative to cement-based augmentation.

## Introduction

Traumatic vertebral compression in the thoracic and lumbar region is common and is usually a painful condition. Vertebral compression caused by minimal trauma is frequently associated with osteoporosis. Annually, a significant number of patients seek medical advice as a result of vertebral compression fractures [1]. Patients with acute and intractable pain that does not respond to analgesic medication frequently require some kind of surgical intervention to relieve the pain. In some healthcare systems, it is obligatory to complete a 4-week course of conservative treatment prior to vertebroplasty being performed [[2], [3]].

Conservative therapy includes the use of analgesics including opiates and nonsteroidal anti-inflammatory medications as well as physiotherapy and the use of a thoracolumbar orthosis. According to several reports, conservative therapy does result in a significant improvement in the majority of cases; however, this improvement often begins only after 6–8 weeks, which delays the commencement of essential exercise therapy and greatly impairs patients’ quality of life. This is supported by the Vertos and the Vertos II studies, which are open-label randomized trials that reported a significant decrease in visual analogue score (VAS) after conservative treatment [[4], [5], [6], [7]]. Percutaneous vertebroplasty and its variants (kyphoplasty and stentoplasty) are used in those cases that do not respond to conservative therapy, because there is often immediate pain relief after the procedure.

The first percutaneous vertebroplasty was performed in France in 1984. The indications for the intervention have multiplied since then; now it is used in the treatment of spinal compression of porotic origin, metastasis and myeloma in the vertebral body, aggressive vertebral hemangioma, osteogenesis imperfecta, vertebral osteonecrosis, traumatic compression fractures, and to strengthen the vertebral bodies before transpedicular screw fixation [[8], [9], [10]]. During the operation, bone cement is introduced into the vertebral body via a minimally invasive percutaneous method. Historically, extra-vertebral spread of bone cement was a significant problem, with an early incidence up to 70%. Although this rate has decreased significantly, the rate of severe complications such as pulmonary embolism and caudal nerve root compression is still significant at between 1% and 6%, and asymptomatic or symptomatic cement leakage remains a persistent procedural risk [1].

Studies have confirmed that the efficiency of pain relief is 75%–80% in these cement-based procedures. Vertebroplasty does prevent further compression in the involved vertebra; however, it is not suitable for the correction of vertebral body height and kyphosis. Some of these challenges were overcome by kyphoplasty, in which a balloon of high-pressure resistance is placed in the vertebral body, filled with contrast material, and bone cement is introduced into the resulting cavity [[11], [12], [13], [14], [15]].

An alternative approach is thermal facet ablation. The main indication for thermal facet ablation is lower back pain caused by osteoarthrosis of the facet joints. The spinal facet joint is innervated by an ascending and descending medial sensory branch of the dorsal ramus of a spinal nerve root. During the procedure, radiofrequency, laser, or cryoablation is used for the denervation of facet joints [[16], [17], [18]]. The most frequently used denervation technique is radiofrequency ablation (RFA), which selectively destroys the afferent sensory nerve fibers with thermal energy. The procedure is associated with a minimal complication profile, primarily limited to transient neuritis or localized pain at the injection site [19], making it an attractive choice compared with more invasive structural procedures. Initially, this intervention was performed exclusively for facet joint osteoarthrosis, but later the indication was extended to include compression vertebral fractures. While previous smaller-scale reports have indicated good initial results for RFA in fracture pain, large-scale, prospective comparative trials between RFA and cement augmentation have been lacking.

This prospective randomized controlled trial presents results accumulated over a 5-year period, comparing vertebroplasty and balloon kyphoplasty against thermal facet ablation in the treatment of vertebral compression fractures at a single center.

## Methods

### Study design and patient population

This study was designed as a single-center, prospective, 2-arm randomized controlled trial comparing radiofrequency thermal facet ablation with vertebral augmentation in patients with painful vertebral compression fractures of the thoracolumbar spine refractory to conservative management. The study protocol was approved by the Institutional Review Board, and all procedures were performed in accordance with the Declaration of Helsinki. Written informed consent was obtained from all patients prior to enrollment and randomization. The trial was prospectively registered at ClinicalTrials.gov.

Eligible patients were adults (≥18 years) presenting with symptomatic vertebral compression fractures in the thoracic or lumbar spine, confirmed by computed tomography (CT), magnetic resonance imaging (MRI), or both. If the history was uncertain, an MRI scan (STIR sequence) was utilized to estimate the age of a fracture by identifying acute bone marrow edema (Fig. 1). Only patients with clinically significant axial pain that persisted despite an adequate course of conservative management were considered for enrollment. Conservative therapy included analgesic medication according to the World Health Organization (WHO) pain ladder, physiotherapy, and the use of a thoracolumbar orthosis.

**Fig. 1:**
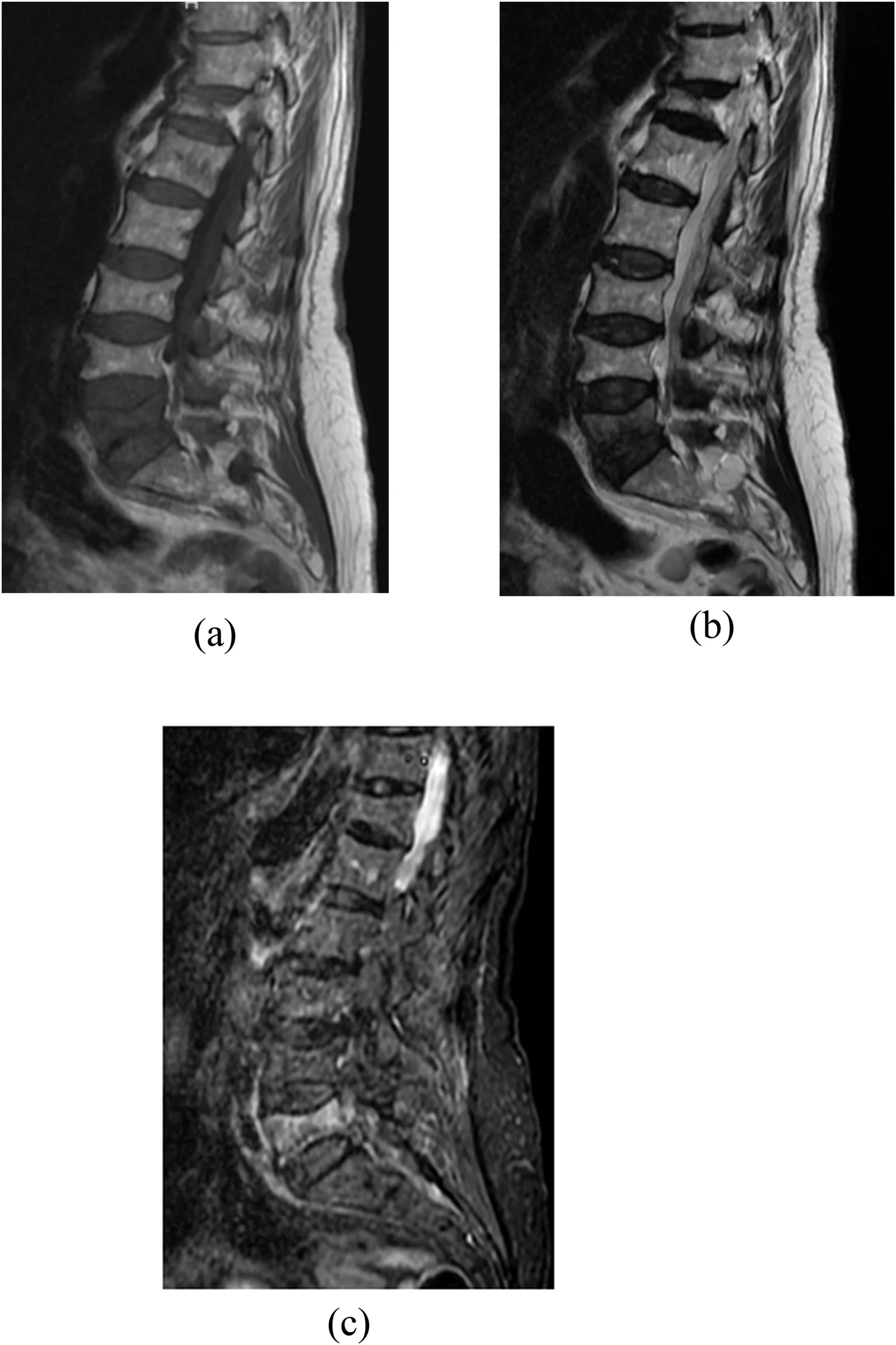
Preoperative sagittal MRI of the lumbar spine demonstrating acute and chronic vertebral compression fractures. (Left) T1-weighted, (Middle) T2-weighted, and (Right) STIR sequences. The hyperintensity of the L5 vertebral body on the STIR image (Right) confirms acute bone marrow edema, establishing the recent nature of the fracture, whereas the old fracture at T12 shows no signal alterations.

The primary inclusion criteria required a radiologically confirmed vertebral compression fracture in the thoracolumbar region, persistent axial spinal pain despite conservative treatment, and the ability to comply with follow-up. Patients were excluded if they had a clinical condition precluding surgical intervention, uncorrected coagulopathy, active systemic infection, suspicion of primary malignant or infectious vertebral pathology, a history of previous radiofrequency ablation or vertebroplasty at the same level within 12 months, or severe neurological deficits requiring urgent decompressive surgery.

Conservative treatment was deemed ineffective if patients reported no improvement in their pain level or if they still experienced significant difficulty walking or performing everyday activities despite a nominal decrease in their pain score [[5], [20]].

### Randomization, blinding, and interventions

Following confirmation of eligibility, patients were randomized in a 1:1 ratio to 1 of 2 treatment arms using a computer-generated random sequence. Group 1 received radiofrequency thermal facet ablation targeted to the facet joints associated with the fractured vertebral level. Group 2 received vertebral augmentation. Within Group 2, the choice between vertebroplasty and kyphoplasty was not randomized but determined by predefined anatomical criteria (eg, degree of collapse, chronicity on MRI).

Due to the surgical nature of the procedures, blinding of the operating surgeons and patients was not feasible. However, to minimize bias, a single-blinded design was adopted for outcome assessment. Postoperative clinical outcomes and VAS scores were recorded at discharge, 2 months, and 6 months by independent clinical administrators fully blinded to the treatment allocation.

Vertebroplasty and kyphoplasty were performed under general anesthesia with standard monitoring and fluoroscopic guidance, utilizing polymethyl methacrylate (PMMA) injected via a transpedicular approach (Fig. 2) [[1], [5], [11], [12], [13], [14], [21], [22]]. Thermal facet ablation was performed under local anesthesia. Radiofrequency neurotomy of the medial branches was executed at 80°C for 1 minute after proper sensory and motor test stimulation (Fig. 3). Finally, the facet joints were infiltrated with 40 mg methylprednisolone [[19], [23], [24], [25]].

**Fig. 2:**
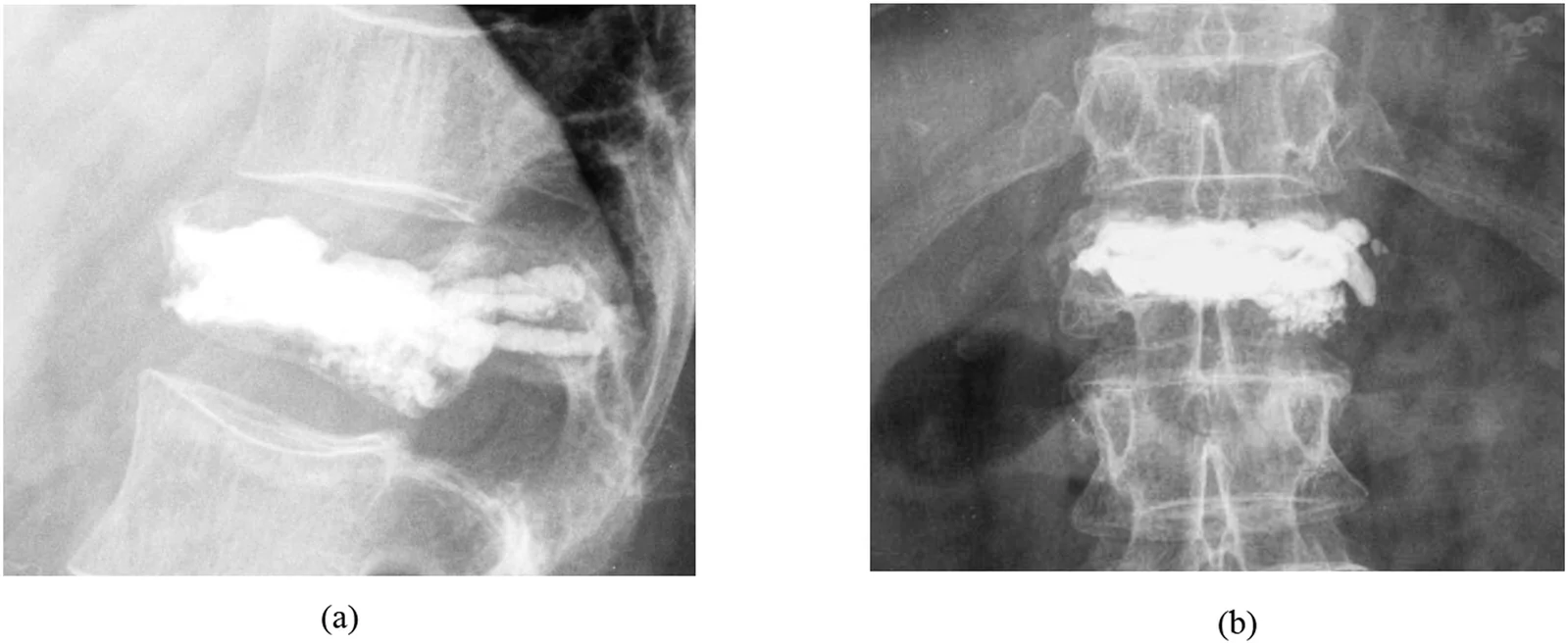
Postoperative radiological evaluation of percutaneous vertebroplasty. (Left) Lateral and (Right) anteroposterior spinal radiographs following L1 vertebroplasty. The radiopaque polymethyl methacrylate (PMMA) bone cement introduced via a transpedicular approach is well-distributed within the vertebral body and pedicular canals.

**Fig. 3:**
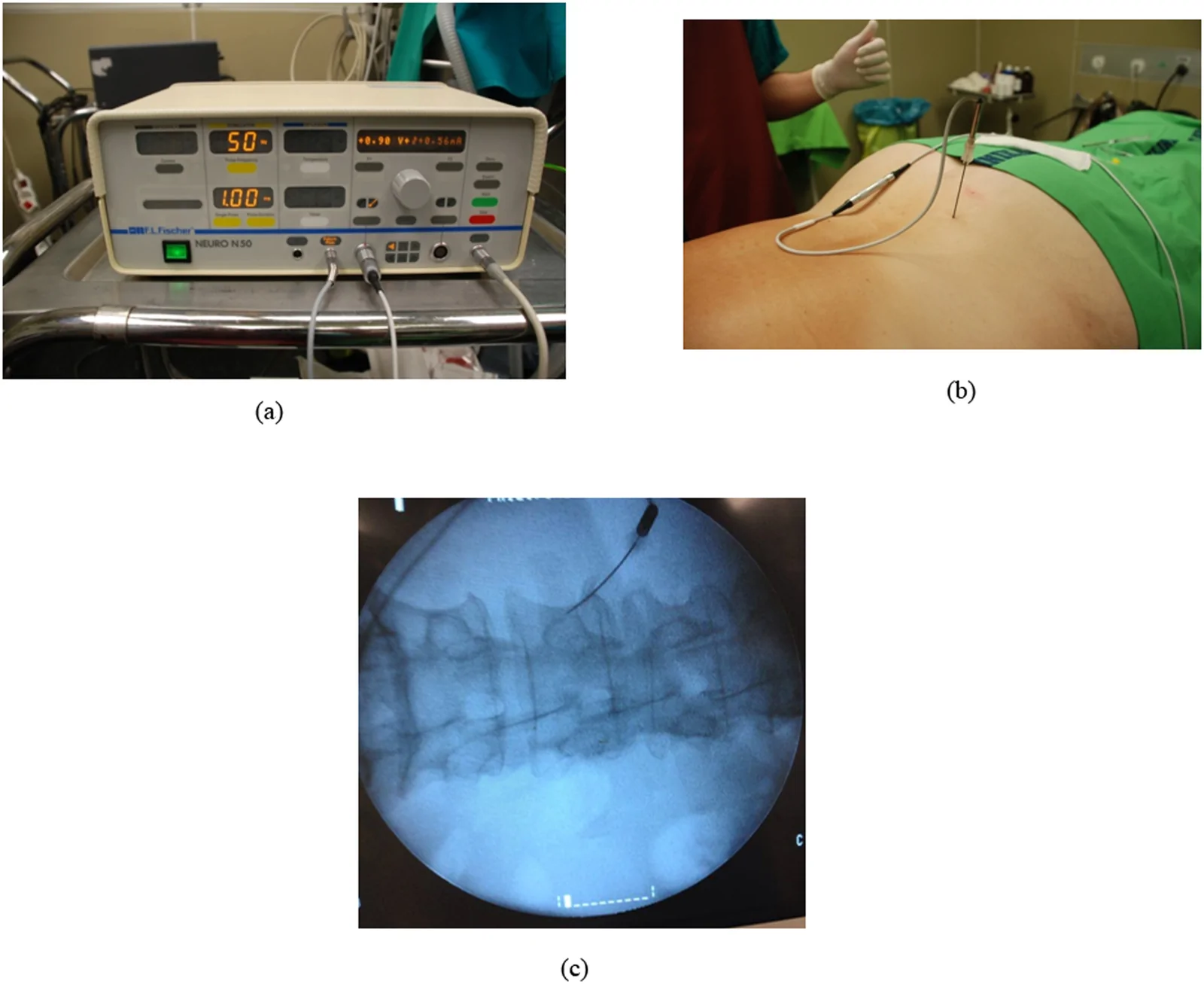
Intraoperative setup and electrode positioning for radiofrequency thermal facet ablation. (Left) The Inomed Neuro N50 Lesion Generator utilized for neurotomy; (Middle) Intraoperative clinical photograph demonstrating the percutaneous electrode placement in local anesthesia; (Right) Intraoperative anteroposterior fluoroscopic image confirming the proper anatomical position of the electrode at the target facet joint segment.

### Outcome measures and statistical analysis

The primary outcome was the reduction in axial spinal pain assessed by the VAS at the 6-month postoperative follow-up [[4], [6]]. Secondary outcomes included procedure-related complications and length of hospital stay (LOS). Continuous variables were summarized as means±standard deviations and categorical variables as percentages. Comparisons between the 2 randomized arms were analyzed using independent-samples *t* tests and chi-square tests, as appropriate. A post-hoc power calculation demonstrated that the final sample size of 535 provided >80% power (*α*=0.05) to detect a clinically meaningful 1.0-point difference in VAS change between the 2 groups.

## Results

### Patient cohort and baseline characteristics

A total of 544 patients met the inclusion criteria and were randomized. During the 6-month follow-up period, 9 patients (1.7%) were lost to follow-up, resulting in a final analyzed cohort of 535 patients (Fig. 4). Of these, 245 patients (45.8%) were allocated to radiofrequency thermal facet ablation (Group 1), and 290 patients (54.2%) were allocated to vertebral augmentation (Group 2), which consisted of 255 vertebroplasties and 35 balloon kyphoplasties (Fig. 5).

**Fig. 4:**
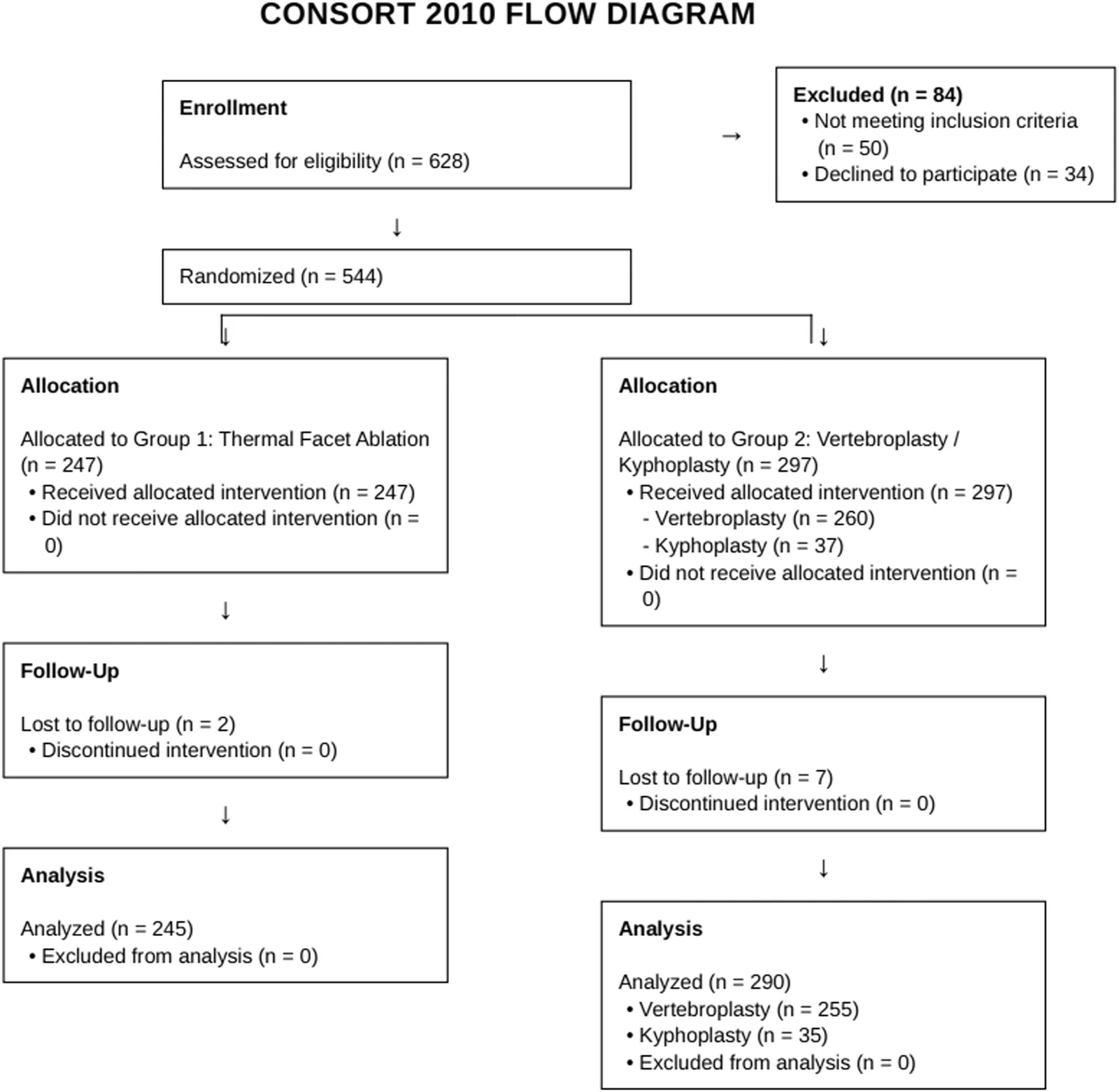
CONSORT 2010 flow diagram. Flowchart illustrating the formal screening, randomized allocation, treatment pathways (Group 1: Thermal facet ablation; Group 2: Vertebral augmentation), 6-month longitudinal follow-up, and final statistical analysis of the study cohort.

**Fig. 5:**
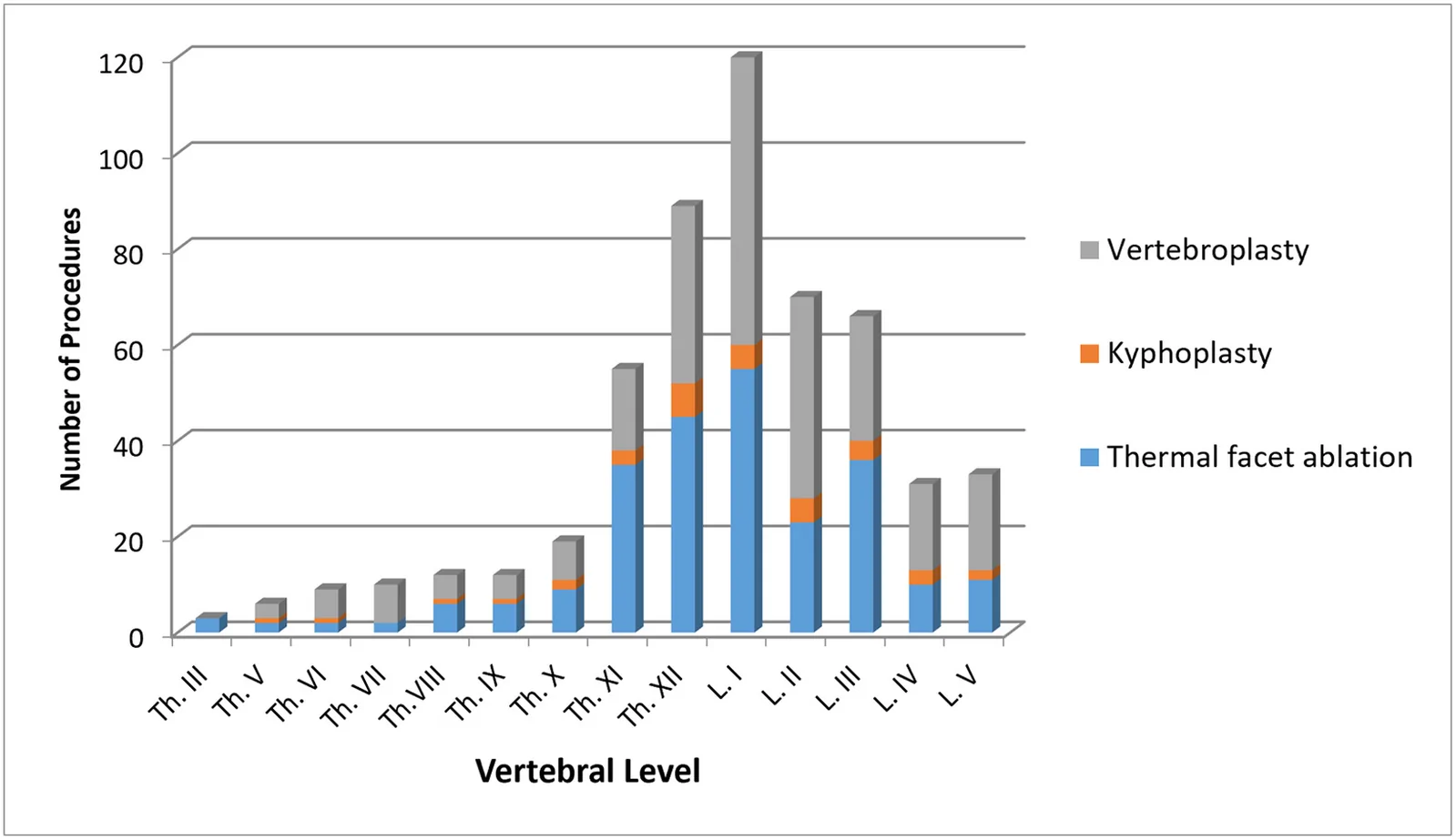
Anatomical distribution of treated vertebral levels and compression fractures. Graphical representation demonstrating that the interventions were predominantly located at the thoracolumbar junction, shifting from T12 to L3, with percutaneous vertebroplasty serving as the most frequent procedure.

Baseline demographic and clinical characteristics were well balanced between the 2 randomized groups (Table 1). The mean age was 64.7±8.2 years for the RFA group and 65.1±8.8 years for the augmentation group. Regarding etiology, 81.6% suffered from osteoporotic fractures in the RFA group and 82.4% in the augmentation group. The mean time from fracture onset to intervention was 48±12 days (RFA) and 51±15 days (augmentation). The duration of active conservative management prior to intervention was 37±14 days and 42±19 days, respectively. The mean preoperative VAS score for axial spinal pain was 7.5±1.2 for RFA and 7.7±1.1 for augmentation, with no statistically significant difference between the study arms at baseline.

**Table 1:** Baseline demographic and clinical characteristics.

Variable	Group 1: Thermal facet ablation (*n*=245)	Group 2: Vertebral augmentation (*n*=290)	p-Value
Mean age (y) mean±SD	64.7±8.2	65.1±8.8	.65
Sex (female/male)	174/71	216/74	.88
Fracture etiology: osteoporotic, *n* (%)	200 (81,6%)	239 (82.4%)	.81
Fracture etiology: traumatic, *n* (%)	45 (18.4%)	51 (17.6%)	.81
Preoperative VAS score, mean±SD	7.5±1.2	7.7±1.1	.42
Time from fracture to procedure (d)	48±12	51±15	.38
Duration of conservative management (d)	37±14	42±19	.31

### Clinical outcomes and pain reduction

At the 6-month follow-up, both RFA and vertebral augmentation produced statistically significant reductions in pain scores relative to baseline. The mean VAS reduction was −5.3 (95% CI: −5.6 to −5.0) in the RFA group and −5.6 (95% CI: −5.9 to −5.3) in the augmentation group. Complete or near-complete pain resolution (VAS 0–1) and substantial pain reduction to mild residual pain (VAS 2–4) were observed at comparable frequencies. Specifically, following RFA, 44% of patients reported VAS 0–1, 27% reported VAS 2–4, and 29% reported moderate to severe residual pain (VAS ≥ 5). Following vertebral augmentation, 28% reported VAS 0–1, 46% reported VAS 2–4, and 26% reported VAS ≥ 5. There was no statistically significant difference in the overall distribution of VAS categories between the randomized groups (p=.35) (Table 2 and Fig. 6).

**Table 2:** Clinical outcomes and complications at 6-month follow-up.

Outcome measure	Group 1: Thermal facet ablation (*n*=245)	Group 2: Vertebral augmentation (*n*=290)	p-Value
Preoperative VAS (mean)	7.5	7.7	.42
6-mo postoperative VAS (mean)	2.2	2.1	.41
Absolute mean change	−5.3	−5.6	.5
95% CI of change	(−5.6 to −5.0)	(−5.9 to −5.3)	–
VAS 0–1 (complete/near complete), *n* (%)	108 (44%)	82 (28%)	.35*
VAS 2–4 (substantial decrease), *n* (%)	66 (27%)	133 (46%)	–
VAS 5–10 (moderate to severe), *n* (%)	71 (29%)	75 (26%)	–
Complications, *n* (%)	0 (0%)	23 (8%)	<.01
Length of hospital stay (d), mean	0.0	1.4	<.01

⁎ The p-value of 0.35 represents the result of the chi-square test comparing the overall distribution of VAS categories (0–1, 2–4, 5–10) between the 2 groups

**Fig. 6:**
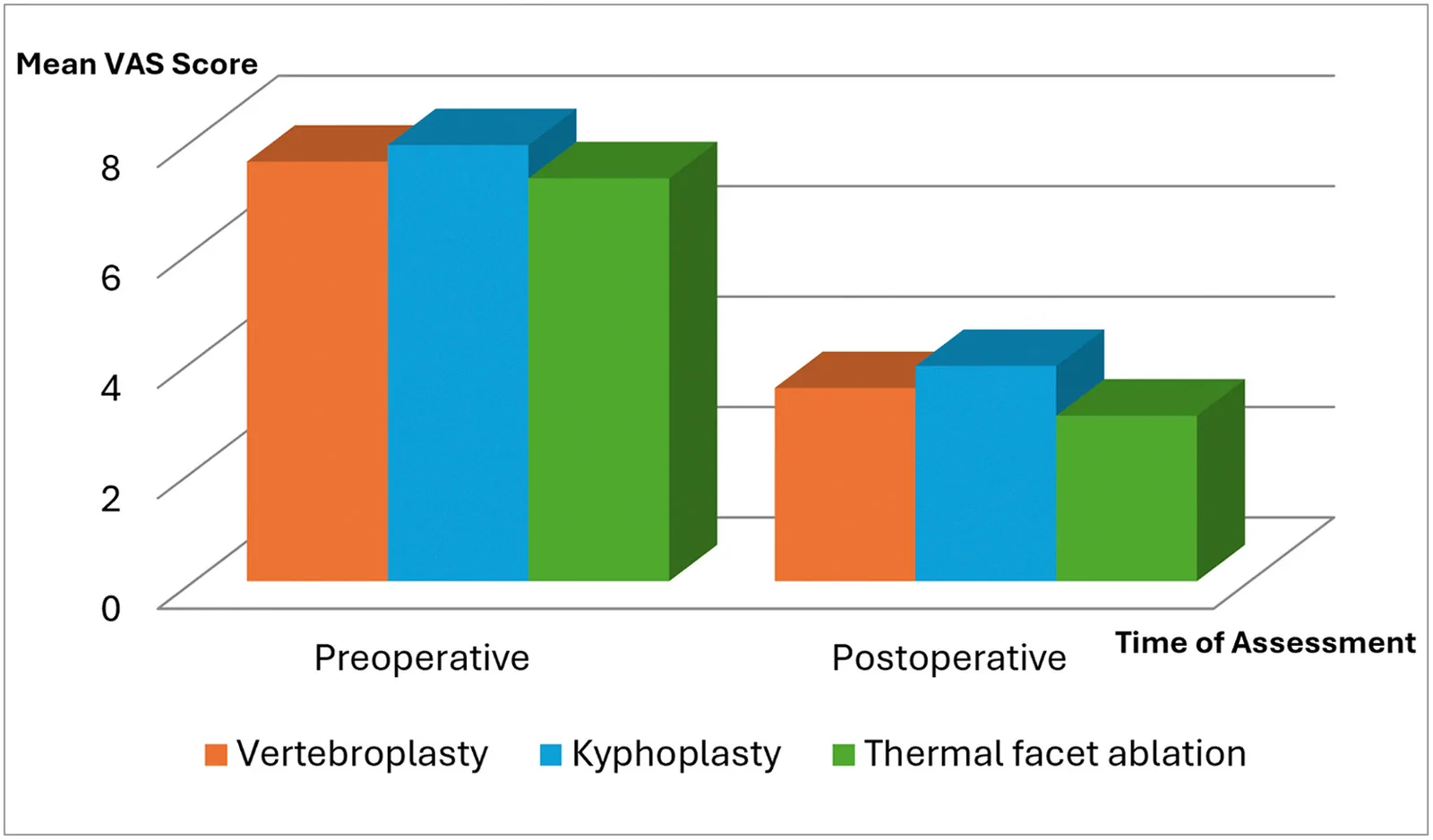
Longitudinal transition and decrease in pain levels based on VAS scores. Clinical pain scores color-coded to contrast baseline preoperative intensity against the documented values at the final 6-month postoperative follow-up for the analyzed treatment arms.

### Complications and length of stay

Perioperative and postoperative complication rates differed between the treatment modalities. In the vertebral augmentation arm, PMMA cement leakage occurred in 23 cases (8%), with 2 patients (0.5%) developing symptomatic radicular pain requiring subsequent posterior decompressive surgery. In contrast, no procedure-related complications were recorded in the RFA arm. Furthermore, the mean length of hospital stay (LOS) was 0.0 days for the RFA cohort, with all patients safely discharged on the day of the procedure, compared to a mean LOS of 1.4 days for the vertebral augmentation cohort (p < .01).

## Discussion

This prospective, single-center, randomized controlled trial demonstrates that radiofrequency thermal facet ablation provides comparable analgesic outcomes to traditional vertebral augmentation as a treatment strategy for painful vertebral compression fractures refractory to conservative management. Both randomized arms achieved robust and sustained reductions in axial spinal pain at the 6-month follow-up, as evidenced by the overlapping 95% confidence intervals for mean VAS change. All 3 interventional techniques—vertebroplasty, kyphoplasty, and thermal facet ablation—proved clinically effective in this setting.

An important clinical distinction emerged regarding the safety profile and resource utilization. While vertebral augmentation was associated with an overall cement leakage rate of 8% and a small but relevant risk of symptomatic complications requiring reoperation, RFA was not associated with any recorded procedure-related complications. The elimination of overnight hospital stays (mean LOS 0.0 vs. 1.4 days) and the avoidance of complication-related readmissions represent potential reductions in healthcare resource utilization, underscoring the outpatient feasibility of the RFA approach.

A notable biomechanical limitation of thermal facet ablation is its inability to restore vertebral body height or correct kyphotic deformity, which balloon kyphoplasty can partially address. However, for patients prioritizing immediate analgesia over structural restoration, this downside is often outweighed by the procedure’s minimal invasiveness. Because the thermal facet ablation protocol combines precise neurotomy of the medial branches via radiofrequency energy with a localized injection of anesthetics and corticosteroids, it successfully targets the secondary arthrogenic and capsular pain components caused by altered spinal mechanics following a fracture.

Current consensus and recent literature reviews support prompt vertebral augmentation within 3 weeks of fracture onset for severe pain but also reinforce that conservative modalities remain a valid initial pathway, with intervention reserved for intractable cases [[21], [26]].

In standard clinical practice, the duration of conservative management ranges widely. Our cohort demonstrated a mean duration of conservative management between 37 and 42 days, establishing a robust conservative threshold before interventional screening. Furthermore, our inclusion of adult patients over 18 allowed for the observation of 96 patients with purely traumatic fractures. Although traditionally reserved for osteoporotic compression, the observed trend suggests that a nonstructural, low-risk analgesic procedure like RFA is also a viable option to expedite rehabilitation in younger, nonosteoporotic trauma patients [[27], [28], [29], [30], [31], [32], [33], [34], [35], [36]].

The primary strength of this study lies in its prospective, randomized controlled design, featuring a large cohort and independent blinded outcome assessment. Limitations include its single-center nature, which may restrict generalizability, and the impossibility of double-blinding the operating surgeons. Long-term outcomes beyond 6 months, such as adjacent-level fracture rates or permanent sagittal alignment changes, were not evaluated and warrant future multi-center investigation.

In conclusion, radiofrequency thermal facet ablation is a highly effective, safe alternative to traditional vertebral augmentation techniques for painful vertebral compression fractures. While achieving comparable clinical pain relief, RFA maintained a zero-complication rate and facilitated immediate, same-day patient discharge. RFA should be considered a robust first-line interventional modality in the contemporary management of vertebral compression fractures.

## Declarations of competing interests

The authors report no conflict of interest concerning the materials or methods used in this study or the findings specified in this article.
